# Supplementary material for: Content-rich biological network constructed by mining PubMed abstracts
Source: BMC Bioinformatics. 2004 Oct 8;5:147. doi: 10.1186/1471-2105-5-147 (PMC528731; doi:10.1186/1471-2105-5-147)
Supplement: Additional File 2 — The original results of the above study (non-essential files are deleted to keep the file size under the limit set by BMC bioinformatics). [file 1471-2105-5-147-S2.bz2 › chilibotAdditionalFile2/dip05/186ID8627325E631/html/ADA_ADORA1.html]

 


 **ADA** and **ADORA1** 
  
Found 67 abstracts in PubMed, retrieved 05.  
 

 What does Google say? 
 PDF only 
| .edu only 

---

- Pol J Pharmacol< MedlineTA>Pol J Pharmacol,   **Neuroprotective role of adenosine in the CNS.** .
  It is well established that in the CNS, endogenous adenosine plays a pivotal role in neurodegeneration.
  A low, nanomolar concentration of adenosine is normally present in the extracellular fluid, but it increases dramatically during enhanced nerve activity, hypoxia or ischemia.
  In these pathological conditions, adenosinergic transmission potentiating agents, which elevate adenosine level by either inhibiting its degradation adenosine deaminase **[ADA]** and kinase inhibitors or preventing its transport, offer protection against ischemic or excitotoxic neuronal damage.
  The directly acting adenosine A1 receptor **[ADORA1]** agonists are known to mediate neuroprotection, mostly by the blockade of Ca2 influx, which results in the inhibition of glutamate release and reduction of its excitatory effects at a postsynaptic level.
  More recent data have shown that antagonists of adenosine A2A receptors markedly reduce cerebral ischemic damage in animal models of focal and global ischemia.
  Moreover, these compounds attenuate the neuronal loss induced by excitatory amino acids EAA .
  A neuroprotective effect of adenosine A2A receptor antagonists was also shown in animal models of Parkinson s disease MPTP, 6 OHDA, methamphetamine .
  Hence, it might be suggested that adenosine A2A receptor antagonists may represent a novel strategy in the therapeutic approach to pathologies characterized by acute or chronic neurodegenerative events, since they not only reverse motor impairment but can act as neuroprotective compunds by promoting cell survival.

  - Pharmacol Biochem Behav, 2002   **Broad spectrum anticonvulsant activity of BW534U87 possible role of an adenosine dependent mechanism.** .
    The novel putative anticonvulsant drug 1 difluorophenyl methyl 1H ,3 triazolo c pyridine 4 amine monohydrochloride BW534U87 effectively reduced seizures induced in rodents by threshold maximal and supramaximal electroshock, electrical kindling, pentylenetetrazole PTZ infusion and by vestibular stimulation in the genetically seizure prone epilepsy like EL mouse.
    The range of animal seizure models in which BW534U87 was effective is consistent with a broad spectrum anticonvulsant profile.
    In the EL mouse, the activity of BW534U87 was partially reversed by predosing with the selective adenosine A1 receptor **[ADORA1]** antagonist 8 cyclopentyl dipropylxanthine DPCPX , suggesting that an adenosine dependent mechanism contributed to the antiseizure activity of the drug.
    BW534U87 inhibited rat brain homogenate adenosine deaminase **[ADA]** activity, thus, raising the possibility that, by blocking the metabolism of endogenous adenosine by this route, BW534U87 limited seizure activity by promoting the inhibitory tone mediated by endogenous adenosine in the brain.
    The seizure protection conferred by the selective adenosine deaminase **[ADA]** inhibitor erythro 9 2 hydroxy 3 nonyl adenine EHNA in EL mice and mice infused with PTZ confirms that inhibition of adenosine metabolism by deamination is an effective antiseizure strategy in these models.

    - Med Sci Monit, 2002   **Depot specific differences in the lipolytic effect of leptin on isolated white adipocytes.** .
      BACKGROUND In the present study we hypothesized that leptin induced lipolytic effects show depot specific differences.
      MATERIAL METHODS Three month old male lean and obese fa fa rats were used.
      Lipolysis was determined in the absence or presence of leptin 0.6 .25 and 62.5 nM together with agents acting at the A1 adenosine receptor of fat cells isolated from both subcutaneous and omental depots.
      The glycerol released into the incubation medium was taken as the lipolytic rate index.
      RESULTS The highest dose of leptin produced a 57.5 7.3% and 70.3 3.8% increase in omental and subcutaneous glycerol release, respectively, compared to the basal lipolytic rate P<0.001 .
      The addition of the three leptin concentrations in the ligand free state produced a significantly greater stimulation of lipolysis in subcutaneous fat cells P=0.0331.
      P=0.0003 and P=0.0015, respectively compared to omental adipocytes.
      Under adenosine A1 receptor **[ADORA1]** agonism and antagonism the same pattern of response was observed between visceral and subcutaneous adipocytes of lean rats.
      Although adenosine deaminase **[ADA]** produced near maximum lipolysis in the adipocytes of lean animals, only half of the maximum lipolytic rate 50.9 3.2% was achieved in fat cells from fa fa rats P=0.0034 .
      Leptin had no effect on the lipolytic activity of adipocytes from either localization in fa fa rats.
      CONCLUSIONS It can be concluded that decreased sensitivity to the lipolytic effect of leptin in omental adipocytes compared to subcutaneous fat cells may underlie, at least in part, the association of visceral fat accumulation with increased co morbidities.

      - J Auton Pharmacol, 2001   **Activation of presynaptic A1 receptors by endogenous adenosine inhibits acetylcholine release in the guinea pig ileum.** .
        1.
        It is well established that presynaptic adenosine A1 receptor **[ADORA1]** activation inhibits acetylcholine ACh release in the guinea pig ileum.
        The present study extends this observation and examines a possible role for endogenous adenosine in modulating cholinergic nerve function.
        2.
        The actions of the adenosine uptake blocker, dipyridamole, the adenosine deaminase **[ADA]** inhibitor, erythro 9 2 hydroxy 3 nonyl adenine EHNA and the A1 receptor antagonist, dipropyl 8 cyclopentylxanthine DPCPX were examined on electrically evoked neurogenic, cholinergic twitch contractions of the guinea pig ileum.
        Some additional studies measuring 3H ACh release were also performed.
        3.
        Adenosine and the selective A1 receptor agonist, 2 chloroadenosine 2 CA , inhibited electrically evoked contractions and, in the case of 2 CA, 3H ACh release.
        The actions were antagonized by DPCPX.
        At low concentrations, dipyridamole and EHNA enhanced the effect of adenosine causing a leftward shift of the concentration response curve.
        In contrast, inhibition induced by 2 CA was unaffected by either dipyridamole or EHNA.
        4.
        When applied alone at higher concentrations, EHNA and dipyridamole produced a concentration dependent suppression of cholinergic neurotransmission.
        In both cases, the effect could be reversed by DPCPX.
        At the same concentration, DPCPX alone produced a small but consistent increase in twitch height and 3H ACh release.
        5.
        The data confirm the existence of inhibitory presynaptic adenosine A1 receptors modulating cholinergic nerve function in the guinea pig ileum and suggests that these receptors can be activated by endogenous adenosine released either as adenosine itself or as an ATP metabolite.
